# Characterization of miRNAs in Milk Small Extracellular Vesicles from Enzootic Bovine Leukosis Cattle

**DOI:** 10.3390/ijms231810782

**Published:** 2022-09-15

**Authors:** Fumi Tsukada, Shigeo Takashima, Yoshiko Wakihara, Yuji O. Kamatari, Kaori Shimizu, Ayaka Okada, Yasuo Inoshima

**Affiliations:** 1 Laboratory of Food and Environmental Hygiene, Cooperative Department of Veterinary Medicine, Faculty of Applied Biological Sciences, Gifu University, Gifu 501-1193, Japan; 2 Division of Genomics Research, Life Science Research Center, Gifu University, Gifu 501-1193, Japan; 3 Institute of Glyco-Core Research (iGCORE), Gifu University, Gifu 501-1193, Japan; 4 The United Graduate School of Drug Discovery and Medical Information Sciences, Gifu University, Gifu 501-1193, Japan; 5 Division of Instrumental Analysis, Life Science Research Center, Gifu University, Gifu 501-1193, Japan; 6 Education and Research Center for Food Animal Health, Gifu University (GeFAH), Gifu 501-1193, Japan; 7 The United Graduate School of Veterinary Sciences, Gifu University, Gifu 501-1193, Japan; 8 Joint Graduate School of Veterinary Sciences, Gifu University, Gifu 501-1193, Japan

**Keywords:** biomarker, bovine leukemia virus, bovine milk, miRNA, small extracellular vesicles

## Abstract

Enzootic bovine leukosis (EBL) is a B-cell lymphosarcoma caused by the bovine leukemia virus (BLV). Most BLV-infected cattle show no clinical signs and only some develop EBL. The pathogenesis of EBL remains unclear and there are no methods for predicting EBL before its onset. Previously, it was reported that miRNA profiles in milk small extracellular vesicles (sEVs) were affected in cattle in the late stage of BLV infection. It raised a possibility that miRNA profile in milk sEVs from EBL cattle could be also affected. To characterize the difference in milk of EBL cattle and healthy cattle, we examined the miRNA profiles in milk sEVs from four EBL and BLV-uninfected cattle each using microarray analysis. Among the detected miRNAs, three miRNAs—bta-miR-1246, hsa-miR-1290, and hsa-miR-424-5p—which were detectable using quantitative real-time PCR (qPCR) and are associated with cancers in humans—were selected as biomarker candidates for EBL. To evaluate the utility of these miRNAs as biomarkers for EBL, their levels were measured using milk that was freshly collected from 13 EBL and seven BLV-uninfected cattle. bta-miR-1246 and hsa-miR-424-5p, but not hsa-miR-1290, were detected using qPCR and their levels in milk sEVs from EBL cattle were significantly higher than those in BLV-uninfected cattle. bta-miR-1246 and hsa-miR-424-5p in sEVs may promote metastasis by targeting tumor suppressor genes, resulting in increased amounts in milk sEVs in EBL cattle. These results suggest that bta-miR-1246 and hsa-miR-424-5p levels in milk sEVs could serve as biomarkers for EBL.

## 1. Introduction

Enzootic bovine leukosis (EBL) is a neoplastic disease; it is a B-cell lymphosarcoma of cattle caused by bovine leukemia virus (BLV), which belongs to the genus *Deltaretrovirus* in the family *Retroviridae* [1]. In BLV infection, most infected cattle show no clinical signs, about one-third develop persistent lymphocytosis (PL), which is characterized as nonmalignant polyclonal B-cell proliferation, and a small percentage develop EBL [2,3,4]. Some European countries have eradicated EBL employing the test-and-slaughter approach for BLV-infected cattle [1,5]. In contrast, in Japan, BLV seroprevalence of dairy and beef cattle was 40.9% and 28.7%, respectively [6], and it is, therefore, difficult to control BLV infection using the test-and-slaughter approach. The number of notified EBL cattle at slaughterhouses and farms has gradually increased in Japan [7] (Supplementary Figure S1). As for diagnosis of BLV infection in cattle, several established techniques are routinely used for the detection of antibodies against BLV and viral nucleic acid, including enzyme-linked immunosorbent assay (ELISA) [8], nested polymerase chain reaction (PCR) [9,10], and quantitative real-time PCR [11]. However, these methods are not able to identify which cow will develop EBL. Moreover, there is no vaccine against BLV, treatment for EBL, or prediction method for the onset of EBL. Currently, it is difficult to control BLV-infected cattle and EBL. Thus, to overcome these difficulties, the characteristics of EBL cattle need to be explored.

Small extracellular vesicles (sEVs) are extracellular vesicles with a diameter in the range of 50–150 nm [12,13,14]. sEVs are present in bodily fluids, such as blood, saliva, and breast milk, in humans [13], and contain nucleic acids, such as messenger RNAs (mRNAs), microRNAs (miRNAs), and proteins [15]. sEVs are critical mediators of intercellular communication and have functional roles in cancers [16], especially miRNAs, which are small non-cording RNAs, which play functional roles in sEVs by regulating the expression of target genes and can serve as noninvasive biomarkers for cancers in humans [13,17]. For example, miR-106b levels are higher in the serum sEVs of patients with lung cancer than in healthy volunteers, and miR-106b enhances the migration and invasion abilities of human lung cancer cells by targeting the tumor suppressor, PTEN, and is a biomarker for lung cancer [18]. The levels of miR-21 and miR-210 in serum sEVs were higher in patients with pancreatic cancer than in those with chronic pancreatitis, and these miRNAs might be potential biomarkers for the diagnosis of pancreatic cancer [19].

Bovine milk sEVs contain 1000 ng of total RNA, of which small RNAs form the major component [20]. Nakanishi et al. [21] reported a possible miRNA biomarker in milk sEVs for cattle at a high risk of BLV transmission with a high proviral load (high copy numbers of integrated BLV genome into the host genome in white blood cells), which is indicative of the late stage of infection before the onset of EBL. Therefore, we expected that the development of EBL also affects miRNA profiles in milk sEVs and some miRNAs might be candidates for use as biomarkers for EBL. We focused on characterization of miRNAs in milk sEVs from EBL cattle. The use of milk, which is easily collected twice a day by milking for exploring prospective biomarkers, is better than the use of blood, which is difficult to collect frequently on large farms and is time consuming.

In this study, we examined the profiles of miRNAs in milk sEVs from BLV-uninfected healthy and EBL cattle using microarray analysis with the aim of exploring the biomarkers for EBL. We evaluated the utility of candidate miRNA biomarkers using freshly collected milk samples from BLV-uninfected and EBL cattle.

## 2. Results

### 2.1. BLV Infection and Clinical Status

Data for BLV infection and hematology of cattle used in the microarray analysis and for evaluating the utility of candidate miRNA biomarkers are summarized in Table 1 and Table 2.

### 2.2. Morphology and Nanoparticle Size Analysis of Milk sEVs

**Figure 1:**
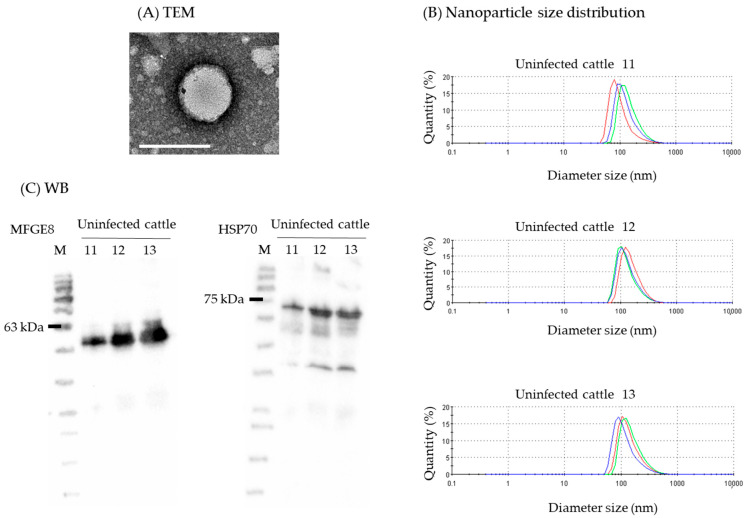
Characterization of isolated milk small extracellular vesicles (sEVs). (**A**) Transmission electron microscopy (TEM) analysis showing the bilayer spherical shape of milk sEVs (Scale bar shows 100 nm). (**B**) Nanoparticle size analysis to determine the size distribution of milk sEVs. Mean peak size of three measurements (red, blue, and green) was observed to be approximately 100 nm in diameter. (**C**) Western blot (WB) analysis using antibodies against sEV surface and internal marker proteins, MFGE8 and HSP70, respectively.

The morphology of isolated milk sEVs was observed using electron microscopy. These sEVs were round with diameters of 100–150 nm (Figure 1A). In the nanoparticle size analysis, the peak of nanoparticle size distribution was approximately 100 nm for all milk sEV samples (Figure 1B). Furthermore, sEV surface marker protein, MFGE8 (53 and 57 kDa), and an internal protein, HSP70 (72–73 kDa), were detected using Western blot (WB) analysis (Figure 1C). These results were consistent with those of previous studies [22,23,24] and indicated the successful isolation of milk sEVs.

### 2.3. Microarray Analysis

To explore candidate miRNA biomarkers for EBL, the miRNAs species and their levels in milk sEVs derived from four uninfected and four EBL cattle were determined using microarray analysis. The obtained data were normalized using the 90-percentile shift, and principal component analysis (PCA) and cluster analysis were performed. These analyses showed that sEV samples from one of the uninfected cows (cow no. 2 in Table 1) could not be separated from sEV samples from EBL cattle (Supplementary Figures S2 and S3), probably due to the quality of this sample or a hidden abnormal clinical status of this cow. Thus, this cow was excluded from subsequent analysis, as described in a previous study [25].

**Figure 2:**
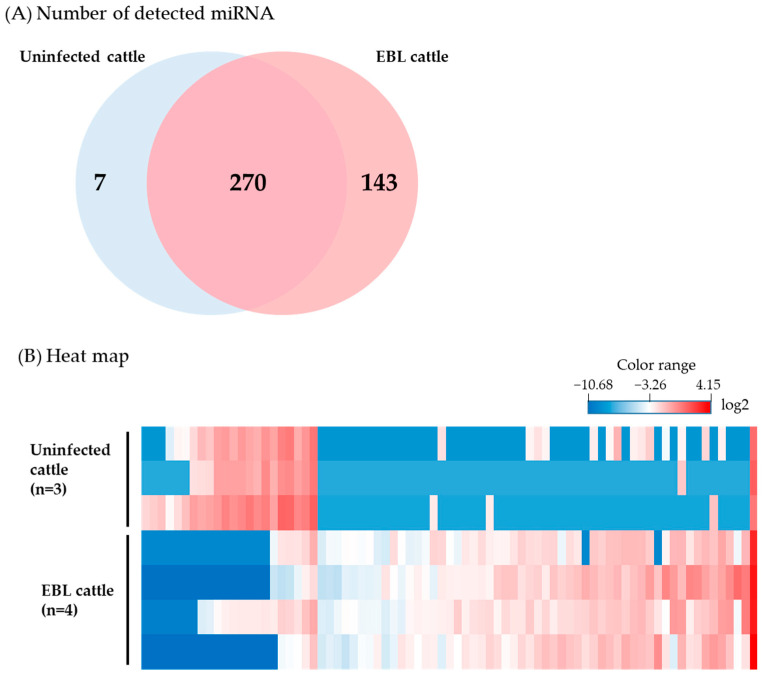
Microarray analysis. (**A**) Numbers of miRNAs detected using the microarray analysis. The number of miRNAs common in the uninfected and enzootic bovine leukosis (EBL) cattle was 270. The number of unique miRNAs in uninfected and EBL cattle were 7 and 143, respectively. These numbers include miRNAs that were detected in only one cattle among three or four cattle. The 143 miRNAs detected only in EBL cattle were not selected for further experiments to explore candidate biomarkers because the coefficient of variation (CV) was > 50%. (**B**) Heatmap of miRNAs detected using the microarray analysis. The microarray data were analyzed using the GeneSpring GX software. Color-coded scale bar represents relative levels of miRNAs (≥5-fold change).

A total of 1399 probes for miRNAs were spotted on an array slide. After normalization and removal of outlier samples, the common number of miRNAs detected in milk sEVs derived from the three uninfected and four EBL cattle was 270, and for the uninfected and EBL cattle were 7 and 143, respectively (Figure 2A). These numbers include miRNAs that were detected in only one cow in each group. The 143 miRNAs detected only in EBL cattle (Figure 2A) were not selected for further experiment for exploration of candidate biomarkers because the coefficient of variation (CV) values was more than 50%.

Next, differentially encapsulated levels of miRNAs in uninfected and EBL cattle were examined as follows. Minute/undetectable miRNAs, which ranked in the lower 20% for all samples in each group, were filtered out, reducing the number to 1280. Thereafter, probes with a CV value < 50% in each group were considered for subsequent analysis, which reduced the number to 1236. Differentially encapsulated levels of miRNAs in the three uninfected and four EBL cattle were identified using the moderated *t*-test [26] with the Benjamini–Hochberg multiple testing correction [27]. miRNAs with a corrected *p*-value < 0.05 were considered significantly fluctuating miRNAs encapsulated in the sEVs, which further reduced the number of miRNAs to 971. Subsequently, the fold-change analysis (≥ 5-fold change) was performed, after which the number of miRNAs was reduced to 77; 55 larger and 22 lower miRNAs than those in the uninfected cattle (Figure 2B). Among these miRNAs, bta-miR-1246 and hsa-miR-1290 were selected as candidate biomarkers for EBL because these were reported to be associated with cancer in humans [28,29]. Furthermore, hsa-miR-424-5p was also selected because it was reported as a biomarker candidate for cattle at a high risk of BLV transmission (late stage of infection, before EBL onset) [21] and, in humans, the levels of hsa-miR-424-5p are reportedly increased in cancer [30]. These three miRNAs were used for further analysis.

### 2.4. Quantitative Real-Time PCR (qPCR) for Detection of Candidate miRNAs in Milk sEVs Used in Microarray Analysis

**Figure 3:**
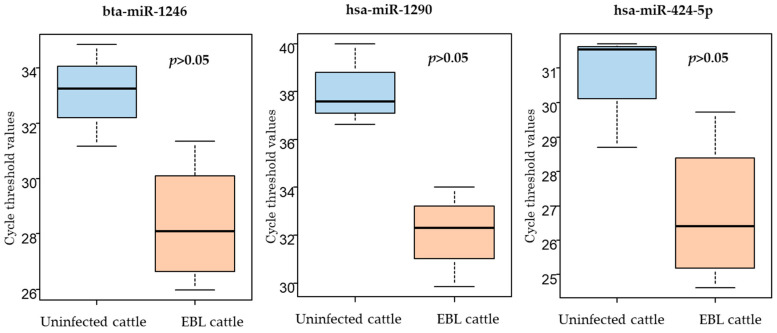
Cycle threshold (Ct) values for qPCR of the three selected miRNAs in milk small extracellular vesicles (sEVs) used in the microarray analysis. The levels of bta-miR-1246, hsa-miR-1290, and hsa-miR-424-5p were higher in milk sEVs from enzootic bovine leukosis (EBL) cattle (*n* = 4) than in those from uninfected cattle (*n* = 3). Mean of Ct values is shown as a horizontal bar in the boxes.

qPCR was performed to confirm whether the three selected miRNAs were applicable as biomarkers for EBL. First, the expression of these three miRNAs was validated using qPCR of milk sEV samples used in the microarray analysis. All the three miRNAs were detected using qPCR, and their levels tended to be higher, but not significantly, in milk sEVs from EBL cattle than in those from uninfected cattle (Figure 3). Thus, bta-miR-1246, hsa-miR-1290, and hsa-miR-424-5p were used for further evaluation of their utility as biomarker candidates for EBL cattle.

### 2.5. qPCR for the Evaluation of the Utility of Candidate miRNA Biomarkers

**Figure 4:**
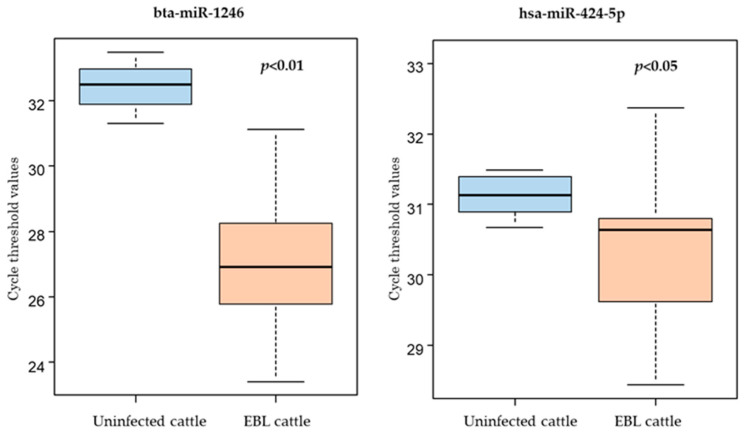
Evaluation of the utility of bta-miR-1246 and hsa-miR-424-5p as biomarkers using freshly collected milk small extracellular vesicles (sEVs) from seven uninfected and 13 enzootic bovine leukosis (EBL) cattle employing qPCR. The levels of bta-miR-1246 and hsa-miR-424-5p were significantly higher in milk sEVs from EBL cattle (*n* = 13) than in those from uninfected cattle (*n* = 7). Mean of cycle threshold values is shown as a horizontal bar in the boxes.

For evaluation of the utility of the three selected miRNAs as candidate biomarkers, qPCR was performed using freshly collected milk sEVs from seven uninfected and 13 EBL cattle (Table 2). The levels of bta-miR-1246 and hsa-miR-424-5p were significantly higher in milk sEVs from EBL cattle than in those from uninfected cattle, in consonance with the results of the microarray analysis (Figure 4). On the contrary, the detection of hsa-miR-1290 was inconsistent due to nonspecific reactions, and it was believed that hsa-miR-1290 would not be a suitable candidate for use as a biomarker under the employed qPCR conditions.

### 2.6. Correlation between the Levels of Candidate miRNA Biomarkers and Several Diagnostic Criteria of EBL

We examined the correlation between the levels of miRNAs and various factors, such as age (Supplementary Figure S4), BLV proviral load (Supplementary Figure S5), total lactose dehydrogenase (LDH) activity (Supplementary Figure S6), LDH isozymes 2 + 3 (Supplementary Figure S7), white blood cell (WBC) count (Supplementary Figure S8), and lymphocyte count (Supplementary Figure S9). Correlations were observed between the levels of bta-miR-1246 and hsa-miR-424-5p and age (*p* < 0.01) (Figure S4) and total LDH activity (*p* < 0.01) (Figure S6). Moreover, the levels of bta-miR-1246 and hsa-miR-424-5p correlated with LDH isozymes 2 + 3 (*p* < 0.01) (Figure S7) and BLV proviral load (*p* < 0.05) (Figure S5), respectively.

## 3. Discussion

In this study, we aimed to explore biomarkers for EBL and focused on the miRNAs present in milk sEVs. We found that the levels of two miRNAs, bta-miR-1246 and hsa-miR-424-5p, were higher in milk sEVs from EBL cattle than in those from BLV-uninfected cattle, suggesting that these miRNAs might be candidate biomarkers for EBL in milk sEVs.

bta-miR-1246 and hsa-miR-1246 are orthologous [31]. In humans, the levels of hsa-miR-1246 were upregulated in blood and this miRNA is one of the biomarkers for hematological malignancies, multiple myeloma [32], and acute myeloid leukemia [33]. Moreover, hsa-miR-1246 suppresses the tumor suppressor gene, DENND2D, and promotes cancer metastasis and invasion in human oral squamous cell carcinoma [28]. Because EBL is a bovine hematological malignancy, bta-miR-1246 may also suppress tumor suppressor genes, such as DENND2D, and may promote the progression of EBL, and could be a candidate biomarker for the development of EBL. However, bta-miR-1246 was reported to be upregulated in blood from heat-stressed cattle [34,35] and in milk, milk sEVs, and mammary gland tissue from mastitis cattle [36,37,38]. Additionally, it was reported that BLV-infection is related with the severity of mastitis [39]. Two of the EBL cattle used in this study (cow nos. 7 and 20 in Table 1 and Table 2) had mastitis and the levels of bta-miR 1246 were higher in milk sEVs from these two cattle than in those from uninfected cattle, but the level was not strongly related with mastitis; the cycle threshold (Ct) values of cow nos. 7 and 20 were 27.30 (the mean of Ct values of the four EBL cattle in Figure 3 was 26.97) and 27.50 (the mean of the 13 EBL cattle in Figure 4 was 25.77), respectively. Therefore, bta-miR-1246 could be a specific biomarker for EBL cattle and further investigation using milk samples from cattle that do not have mastitis is needed. Extensive analysis is also required using milk sEVs from age-matched uninfected and persistent lymphocytosis (PL) BLV-infected cattle.

In this study, a microarray slide was designed with bovine and human miRNA probes, and hsa-miR-424-5p, one of the human miRNAs, was selected as a biomarker candidate for EBL cattle. Human and bovine miRNA sequences selected for the exploration of candidate biomarkers are shown in Table S1. Because the sequence of bta-miR-424-5p is similar to that of hsa-miR-424-5p, the latter might be detected in the microarray analysis. In humans, hsa-miR-424-5p was reported to be upregulated in laryngeal squamous cell carcinoma and promoted proliferation, migration, and invasion by targeting the tumor suppressor, CADM1 [40]. Moreover, hsa-miR-424-5p was upregulated in gastric cancer and promoted the proliferation of gastric cancer cells by targeting Smad3 through the TGF-β signaling pathways [30]. Therefore, miR-424-5p could also be associated with the progression of EBL by suppressing tumor suppressor mRNA to promote proliferation, migration, and invasion of B-cell lymphoma cells. Additionally, because hsa-miR-424-5p levels are correlated with the total activity of LDH, which is released as cells die and is elevated in hematological malignancies including EBL [41,42], the expression of hsa-miR-424-5p might be upregulated when the number of lymphoma cells is increased in the host. Thus, the levels of hsa-miR-424-5p in milk sEVs from EBL cattle might also be higher than in those from uninfected cattle. Considered together with the results of a previous study, wherein the levels of hsa-miR-424-5p were found to be higher in milk sEVs from cattle at a high risk of BLV transmission (late stage of infection, before the EBL onset) than in those from uninfected cattle [21], our results suggest that hsa-miR-424-5p may be a biomarker that can be used to diagnose the progression of EBL before macroscopic tumors are formed.

We considered two reasons for an increase in the levels of bta-miR-1246 and hsa-miR-424-5p in milk sEVs from EBL cattle. First, sEVs containing bta-miR-1246 and hsa-miR-424-5p may be released from lymphoma and may play a role in tumor metastasis to neighboring lymph nodes because the primary tumor releases sEVs to prepare metastatic sites in rats [43]. bta-miR-1246 and hsa-miR-424-5p in sEVs may promote metastasis by targeting tumor suppressor genes in EBL, as mentioned above. Second, mammary epithelial cells released these miRNAs in response to infection and inflammation and sEVs seem to play crucial roles by carrying inflammatory modulators, such as miRNAs [44]. In this study, target mRNAs of miRNAs for biomarker candidates were not investigated, and further analysis in this regard is warranted to decipher functions of the two candidate miRNA biomarkers in EBL. Moreover, further studies, such as comparison of miRNA profiles in milk sEVs with those in blood and lymph nodes and confirmation of miRNA functions in cells, are needed to prove this hypothesis.

In view of the observed correlations of the levels of bta-miR-1246 and hsa-miR-424-5p with EBL diagnostic criteria which were previously reported, these miRNAs might turn out to be novel biomarkers for EBL. Further studies on these miRNAs are required in combination with previously reported criteria for EBL or pre-EBL, such as miRNA [21], mRNA [45,46], and protein [47] biomarkers, and the clinical/hematological/virological status. Because milk is easier to collect than blood, it can be tested frequently to detect cattle immediately before the onset of EBL. However, since this study used post-onset EBL cattle samples, further investigation is needed using pre-onset samples for the evaluation of the utility of these miRNAs as predictive biomarkers.

## 4. Materials and Methods

### 4.1. Animals and Diagnosis

Blood and milk samples were collected from 11 BLV-uninfected healthy and 17 EBL Holstein dairy cattle (Table 1 and Table 2). Samples from BLV-uninfected cattle were collected at the dairy farm of Gifu University (Gifu, Japan) as control cattle samples, and samples from EBL cattle were collected by veterinarians at livestock clinics in Hokkaido and Gifu, a meat hygiene inspection center in Aichi, or at livestock hygiene service centers in Kyoto and Nagasaki. At the livestock clinics and livestock hygiene service centers, EBL cattle were diagnosed based on the manual of Livestock Insurance of National Agricultural Insurance Association [48]. At the meat hygiene inspection center, EBL cattle were diagnosed based on the New Meat Hygiene Inspection Manual [49]. All procedures used in this study were approved by the Gifu University Animal Care and Use Committee (approval numbers 17046 and 2019-234).

### 4.2. Hematology

Blood samples collected from cattle were directly aliquoted into vacuum blood collection tubes, with or without heparin (VP-H070K or VP-AS076K, Terumo, Tokyo, Japan). Total WBCs and lymphocyte counts were measured using a Celltac α MEK-6550 (Nihon Kohden, Tokyo, Japan). Lymphocytosis was checked by determining the lymphocyte counts and age, based on the leukosis-key of the European Community (Key of EC), which is one of the detecting methods for PL cattle [50].

#### 4.2.1. Detection of Serum Antibodies against BLV

Serum was separated from blood by centrifugation at 1350× *g* for 15 min at 25 °C in an R3S rotor using a Himac CR20GII centrifuge (Hitachi Koki, Tokyo, Japan). Levels of anti-BLV antibodies in the serum were measured using an anti-BLV antibody enzyme-linked immunosorbent assay (ELISA) kit (JNC, Tokyo, Japan), according to the manufacturer’s instructions.

#### 4.2.2. DNA Extraction from WBCs

Plasma was removed from 1.3 mL anticoagulated blood by centrifugation. For hemolysis, 1.0 mL of 0.83% NH_4_Cl and 0.01% EDTA in distilled water were added to hemocytes and the suspension was vortexed. WBCs were separated by centrifugation at 600× *g* for 10 min at 25 °C in a TMA-29 rotor using an MX-307 centrifuge or a TMA-29II rotor using an MX-301 centrifuge (Tomy Seiko, Tokyo, Japan). The supernatant was discarded, and the pelleted WBCs were washed with phosphate-buffered saline (PBS). The washed WBCs were separated by centrifugation at 600× *g* for 5 min at 25 °C and the supernatant was discarded. The separated WBCs were suspended in 200 µL of PBS. Total DNA was extracted from WBCs using the DNeasy Blood and Tissue Kit (69506, Qiagen, Hilden, Germany) for detection of BLV provirus and for measurement of the proviral load.

#### 4.2.3. Detection of BLV Provirus Using Nested Polymerase Chain Reaction (PCR)

Nested PCR for detecting the pX [9] or envelope [10] region of BLV in DNA extracted from WBCs was performed using the GoTaq Hot Start Green Master Mix (M512C, Promega, Madison, WI, USA). The thermal cycling conditions were as follows: 94 °C for 9 min, followed by 25 cycles of 94 °C for 45 s for denaturation, 62 °C for 30 s for annealing, and 72 °C for 30 s for amplification, and a final elongation at 72 °C for 4 min. PCR products were electrophoresed on 1% agarose gel and were visualized using ethidium bromide staining.

#### 4.2.4. Measurement of BLV Proviral Load Using Quantitative Real-Time PCR (qPCR)

The amount of BLV proviral DNA (copies/10^5^ WBCs) was measured using qPCR with a CoCoMo-BLV Primer/Probe (A803, RIKEN Genesis, Tokyo, Japan), according to the manufacturer’s instructions. Hematology test, detection of serum antibodies against BLV, and measurement of BLV proviral load were conducted by Gifu Chuo Livestock Hygiene Service Center (Gifu, Japan).

#### 4.2.5. LDH Analysis

LDH isozymes in the serum or plasma were measured using a Hydrasys 2 Scan (Sebia, Paris, France) with HYDRAGEL 7 ISO-LDH (Sebia), by a clinical laboratory testing company, Fujifilm VetSystems (Tokyo, Japan).

### 4.3. Isolation and Characterization of Milk sEVs

Isolation and purification of milk sEVs were carried out as previously described [23,45,46,47,51], with slight modifications. Briefly, after removing the milk fat by centrifugation at 2000× *g* for 20 min using an A2506 centrifuge (Kubota, Tokyo, Japan), defatted milk was preheated at 37 °C for 10 min. For efficient isolation of milk sEVs, acetic acid was added (final concentration, 1%) to the defatted milk and casein was removed by centrifugation at 5000× *g* for 20 min. The whey was filtered using 1.0, 0.45, and 0.2 μm pore-size filters (GA-100, C045A047A, and C020A047A, Advantec, Tokyo, Japan).

According to the Minimal Information for Studies of Extracellular Vesicles 2018 (MISEV2018) guidelines [52], the isolated milk sEVs were characterized biophysically using transmission electron microscopy (TEM), nanoparticle size analysis, and western blot analysis. For observing milk sEVs using TEM, whey was ultracentrifuged at 100,000× *g* for 1 h at 4 °C in a P40ST swing rotor. The pellets were suspended in 2 mL of PBS, layered on the top of a linear sucrose-density gradient (SDG) solution (3 mL each of 10%–20%–40% in distilled water, *w/v*), and ultracentrifuged at 200,000× *g* for 18 h at 4 °C in a P40ST swing rotor. Thereafter, 0.9 mL of each gradient fraction was collected from the top of the tube and numbered from 1 to 12. The SDG fraction no. 12 was diluted with 10 mL of 0.1 µm-filtered water and ultracentrifuged again at 100,000× *g* for 1 h at 4 °C in a P40ST swing rotor. The pellet was suspended in 100 µL of 0.1 µm-filtered water and collected in another tube as an sEV suspension. The sEV suspension was diluted 1:100 with 0.1 μm-filtered distilled water and applied onto glow-discharged polyvinyl butyral support films on copper grids (U1011, EM Japan, Tokyo, Japan). The grids were stained with phosphotungstic acid, and excess solution was removed with filter paper. The dried grids were examined using a JEM-2100F electron microscope (JEOL, Tokyo, Japan) at 200 kV. For nanoparticle size analysis of milk sEVs, whey was ultracentrifuged at 100,000× *g* for 1 h at 4 °C in a P40ST swing rotor, and sEV pellet was suspended in 150 µL of 0.1 µm-filtered water. The sEV suspension was diluted 1:100 with 0.1 µm-filtered water, followed by filtration with a 0.22 µm filter and the nanoparticle size distribution was analyzed using a Zetasizer Nano ZS nanoparticle analyzer (Malvern Panalytical, Worcestershire, UK). Isolated milk sEVs were confirmed by detecting the sEV surface and internal marker proteins, MFGE8 and HSP70, using western blot analysis, as described previously [23,51]. Anti-MFGE8 monoclonal antibody (1:10,000, clone 6F11, a kind gift from Dr. Tsukasa Matsuda, Fukushima University, Japan) [53] and anti-HSP70 monoclonal antibody (1:100, ADI-SPA-820, Enzo Life Science, Farmingdale, NY, USA) were used as primary antibodies and anti-mouse IgG, HRP-linked antibody (1:2000, #7076, Cell Signaling Technology, Danvers, MA, USA) was used as a secondary antibody.

### 4.4. RNA Extraction from Milk sEVs

For exploration of biomarkers for EBL cattle employing microarray analysis, raw milk samples were collected from four uninfected and four EBL cattle (Table 1). For microarray analysis, 30 mL whey was ultracentrifuged at 100,000× *g* for 1 h at 4 °C in a P40ST swing rotor. A total of 30 mL whey was aliquoted into three tubes (10 mL/tube) and was used for the first ultracentrifugation. Whey that was less than 30 mL was adjusted to 30 mL with PBS. After the first ultracentrifugation, the supernatant was discarded, and each pellet from 30 mL whey was suspended in 1 mL PBS and was collected in another tube for the second ultracentrifugation. The pellet obtained by the second ultracentrifugation was suspended in 200 µL of PBS as sEVs and used for further analyses or stored at −80 °C until use. Exosomal RNA was extracted using the miRNeasy Serum and Plasma Kit (217184, Qiagen), according to the manufacturer’s instructions. Before microarray, the quality of extracted RNA was determined using a 2100 Agilent Bioanalyzer (Agilent Technologies, Santa Clara, CA, USA) with an RNA 6000 nano kit (5067-1511, Agilent Technologies) and concentration of extracted RNA was determined using a QuantiFluor RNA System (E3310, Promega).

Next, to evaluate the utility of miRNA biomarker candidates selected using microarray analysis, raw milk samples were collected from seven uninfected and 13 EBL cattle for qPCR (Table 2). Ten milliliter whey was ultracentrifuged at 100,000× *g* for 1 h at 4 °C in a P40ST swing rotor, and the sEV pellet, thus obtained, was suspended in 200 µL of PBS. Exosomal RNA was extracted using the miRNeasy Serum and Plasma Kit. Before qPCR, concentration of extracted RNA was determined using a NanoDrop Lite (Thermo Fisher Scientific).

### 4.5. Microarray Analysis

For microarray analysis, Sure Print G3 Custom miRNA 8×60k (G4871A-#085798, Agilent Technologies), which contains probes for 1399 miRNAs including 786 bovine and 613 human miRNAs on the slide, was used. Hybridized microarray slides were scanned, and fluorescence intensities were measured using an Agilent G2565C microarray scanner (Agilent Technologies). The obtained data were analyzed with the GeneSpring GX Software (Agilent Technologies). The data were normalized by 90-percentile shift according to the manufacturer’s instructions and moderated *t*-test with Benjamini–Hochberg method [27] for multiple testing correction, principal component analysis (PCA) [54], and cluster analysis were performed using the GeneSpring GX Software. The corrected *p*-value cut-off was 0.05.

### 4.6. Quantification of miRNAs in Milk sEVs Using qPCR

Quantification of miRNAs in milk sEVs was carried out using the miRCURY LNA RT Kit (339340, Qiagen) and miRCURY LNA SYBR Green PCR Kit (339346, Qiagen). Briefly, 40 ng of total RNA extracted from milk sEVs was reverse transcribed in 10 µL reaction solution and incubated for 60 min at 42 °C, followed by a further incubation at 95 °C for 5 min to inactivate the reverse transcriptase enzyme. Primers for bta-miR-1246 (YP00205630, Qiagen), hsa-miR-1290 (YP02118634, Qiagen), and hsa-miR-424-5p (YP00204736, Qiagen) were contained in miRCURY LNA miRNA PCR Assay components (339306, Qiagen). For qPCR, cDNA was diluted at 1:30 by adding RNase-free water, and 3 µL of diluted cDNA was used in total volume of the reaction mixture. qPCR was performed using a StepOne Plus analytical thermal cycler (Applied Biosystems, Foster City, CA, USA). The thermal cycling program was as follows: 95 °C for 2 min for initial denaturation, followed by 40 cycles of 95 °C for 10 s for denaturation and 56 °C for 1 min for annealing and extension. Although stably encapsulated miRNAs in milk sEVs should be used as reference miRNAs for the normalization of encapsulation levels of miRNAs, such suitable miRNAs in milk sEVs have not been identified, as yet. Therefore, the amounts of extracted RNA were always adjusted to 40 ng for reverse transcription. After amplification, melt curve analysis was performed to validate the specificity of the reactions. The miRNA amount of undetermined sample was calculated with Ct value = 40 considered to be below the limit of detection.

### 4.7. Statistical Analysis

The data were analyzed for statistical significance using the Mann–Whitney *U*-test. The corrected *p*-value cut off was 0.05.

## 5. Conclusions

The levels of bta-miR-1246 and hsa-miR-424-5p in milk sEVs from EBL cattle were higher than those from uninfected cattle. These two miRNAs could be possible biomarkers for EBL. This study may contribute to the further exploration of predictive biomarkers for EBL cattle that might aid in the control of EBL.

## Figures and Tables

**Table 1:** BLV infection and clinical status of cattle used in microarray analysis *^1^.

Cow No.	Age *^2^ (Month)	ELISA *^3^ Antibody	Nested PCR	Proviral Load *^4^ (/10⁵WBCs)	WBC *^5^ (/μL)	Lymphocyte (/μL)	Total LDH *^6^ (IU/L)	LDH Isozyme (%)						Key of EC *^7^	Sample Collection *^8^
								1	2	3	2 + 3	4	5		
Uninfected cattle															
1	66	−	−	NT	7100	3500	1233	69.1	18.4	7.8	26.2	1.8	2.9	-	A
2	81	−	−	NT	5400	2800	1009	63.9	20.2	10.9	31.1	3.7	1.3	-	A
3	34	−	−	NT	8600	4200	1222	66.8	19.4	9.7	29.1	3	1.1	-	A
4	55	−	−	NT	4800	2000	1246	68.4	17.8	9.6	27.4	3.1	1.1	-	A
EBL cattle *^9^															
5	84	+	+	58,933	13,200	1600	1800	38.5	35.9	18.6	54.5	4.6	2.4	-	B
6	59	+	+	132,721	over ^*10^	NT	5439	30.7	32.1	17.6	49.7	6.2	13.4	NT	C
7	65	+	+	16,696	12,700	7100	1376	37.3	33.2	21.1	54.3	6.4	2	+	C
8	88	+	+	54,024	49,800	7700	2095	35.1	27.3	19.2	46.5	6.3	12.1	+	C

+, positive; −, negative; NT, not tested; *^1^ BLV, bovine leukemia virus; *^2^ age at blood sampling; *^3^ ELISA, anti-BLV antibody enzyme-linked immunosorbent assay; *^4^ measured using a CoCoMo-BLV Primer/Probe (copies/10^5^ WBCs); *^5^ WBC, white blood cell; *^6^ LDH, lactate dehydrogenase; *^7^ key of EC, leukosis-key of the European Community; *^8^ blood and milk samples were collected by veterinarians at A, B, or C. A, a dairy farm at Gifu University; B, NOSAI Douo in Hokkaido; C, Toyohashi City Meat Hygiene Inspection Center in Aichi; *^9^ EBL, enzootic bovine leukosis; *^10^ over, >60,000/μL.

**Table 2:** BLV infection and clinical status of cattle used for evaluation of candidate miRNA biomarkers using qPCR *^1^.

Cow No.	Age *^2^ (Month)	ELISA *^3^ Antibody	Nested PCR	Proviral Load *^4^ (/10⁵WBCs)	WBC *^5^ (/μL)	Lymphocyte (/μL)	Total LDH *^6^ (IU/L)	LDH Isozyme (%)						Key of EC *^7^	Sample Collection *^8^
								1	2	3	2 + 3	4	5		
Uninfected cattle															
9	28	−	−	NT	6100	3200	1327	71.1	15.9	7.4	23.3	3.6	2	−	A
10	29	−	−	NT	9100	4400	1080	61.8	21.5	11.9	33.4	3.6	1.2	−	A
11	31	−	−	NT	4800	2100	1331	69.9	17.1	9.4	26.5	1.8	1.8	−	A
12	77	−	−	NT	6000	3100	1183	60.7	21.6	12	33.6	3.9	1.8	−	A
13	42	−	−	NT	8600	4200	1222	66.8	19.4	9.7	29.1	3	1.1	−	A
14	37	−	−	NT	5400	2400	1304	65.5	18.3	10.2	28.5	3.8	2.2	−	A
15	53	−	−	NT	5400	2700	1190	72.7	14.9	6.9	21.8	2.1	3.4	−	A
EBL *^9^ cattle															
16	100	+	+	32,670	10,700	6100	1434	49.4	31.8	14.8	46.6	3.1	0.9	±	C
17	73	+	+	90,266	21,900	15,600	5000	17.5	12.5	5.7	18.2	3.9	60.4	+	C
18	92	+	+	58,096	5400	2700	4525	36.2	39.4	19.5	58.9	3.4	1.5	−	C
19	99	+	+	45,953	8900	5000	2128	35	29.1	13.9	43	8.5	3.5	±	C
20	44	+	+	95,951	14,800	9700	3134	33	17.8	11.9	29.7	6.9	30.4	+	C
21	77	+	+	32,882	13,200	1600	3362	42.3	27.6	17.2	44.8	6	6.9	−	C
22	68	NT	+	8557	7000	3100	1536	35.4	30	23	53	8.7	2.9	−	C
23	60	+	+	54,442	13,600	7700	1404	56.3	23.9	11.4	35.3	5.1	3.3	+	B
24	48	+	+	212	12,600	7600	1937	51.3	25.8	12.9	38.7	5.6	4.4	+	B
25	62	NT	+	28,824	7200	4700	1980	47.6	33.6	12.2	45.8	3.4	3.2	−	B
26	100	+	+	95,092	20,500	8900	3171	41	38.3	16.1	54.4	3.6	1	+	D
27	72	+	+	58,203	over *^10^	NT	2655	30	36.7	24.9	61.6	7.4	1	NT	E
28	55	+	+	2898	over	NT	1471	38.9	19.5	19.8	39.3	12.4	9.4	NT	F

+, positive; −, negative; NT, not tested; *^1^ BLV, bovine leukemia virus; *^2^ age at blood sampling; *^3^ ELISA, anti-BLV antibody enzyme-linked immunosorbent assay; *^4^ measured using a CoCoMo-BLV Primer/Probe (copies/10^5^ WBCs); *^5^ WBC, white blood cell; *^6^ LDH, lactate dehydrogenase; *^7^ key of EC, leukosis-key of the European Community; *^8^ blood and milk samples were collected by veterinarians at facilities A, B, or C. A, a dairy farm at Gifu University; B, NOSAI Douo in Hokkaido; C, Toyohashi City Meat Hygiene Inspection Center in Aichi; D, NOSAI Gifu in Gifu; E, Chutan Livestock Hygiene Service Center in Kyoto; F, Kennan Livestock Hygiene Service Center in Nagasaki; *^9^ EBL, enzootic bovine leukosis; *^10^ over, >60,000/μL.

## Data Availability

The data presented in this study are available within the article and in the Supplementary Material.

## Supplementary Materials

The following supporting information can be downloaded at: https://www.mdpi.com/article/10.3390/ijms231810782/s1.
