# Supplementary material for: Characterization of miRNAs in Milk Small Extracellular Vesicles from Enzootic Bovine Leukosis Cattle
Source: Int J Mol Sci. 2022 Sep 15;23(18):10782. doi: 10.3390/ijms231810782 (PMC9503721; doi:10.3390/ijms231810782)
Supplement: Supplementary file 1 [file ijms-23-10782-s001.zip › ijms-1895165-supplementary.pdf]

Table S1. miRNA sequence

miRNA※1	Sequence※2	Active sequence in microarray probe※3
bta-miR-1246	AAUGGAUUUUUGGAGCAGG	CCTGCTCCAAAAATCC
hsa-miR-424-5p	CAGCAGCAAUUCAUGUUUUGAA	TTCAAAACATGAATTGCTGCTG
bta-miR-424-5p	CAGCAGCAAUUCAUGUUUUGA	TCAAAACATGAATTGCTGC
hsa-miR-1290	UGGAUUUUUGGAUCAGGGA	TCCCTGATCCAAAAATCC
bta-miR-1290	Unidentified	Not contained

※1 qPCR for bta-miR-1246, hsa-miR-424-5p, and hsa-miR-1290 were conducted using primers contained in miRCURY LNA miRNA PCR assay (339306, Qiagen), YP00205630, YP00204736, and YP02118634, respectively.

※2 Sequence information of mature miRNAs was obtained from miRBase (http://www.mirbase.org/) (accessed on 7 October, 2021).

※3 An active sequence was contained in the custom array slide (G4871A-#085798, Agilent) used in this study.

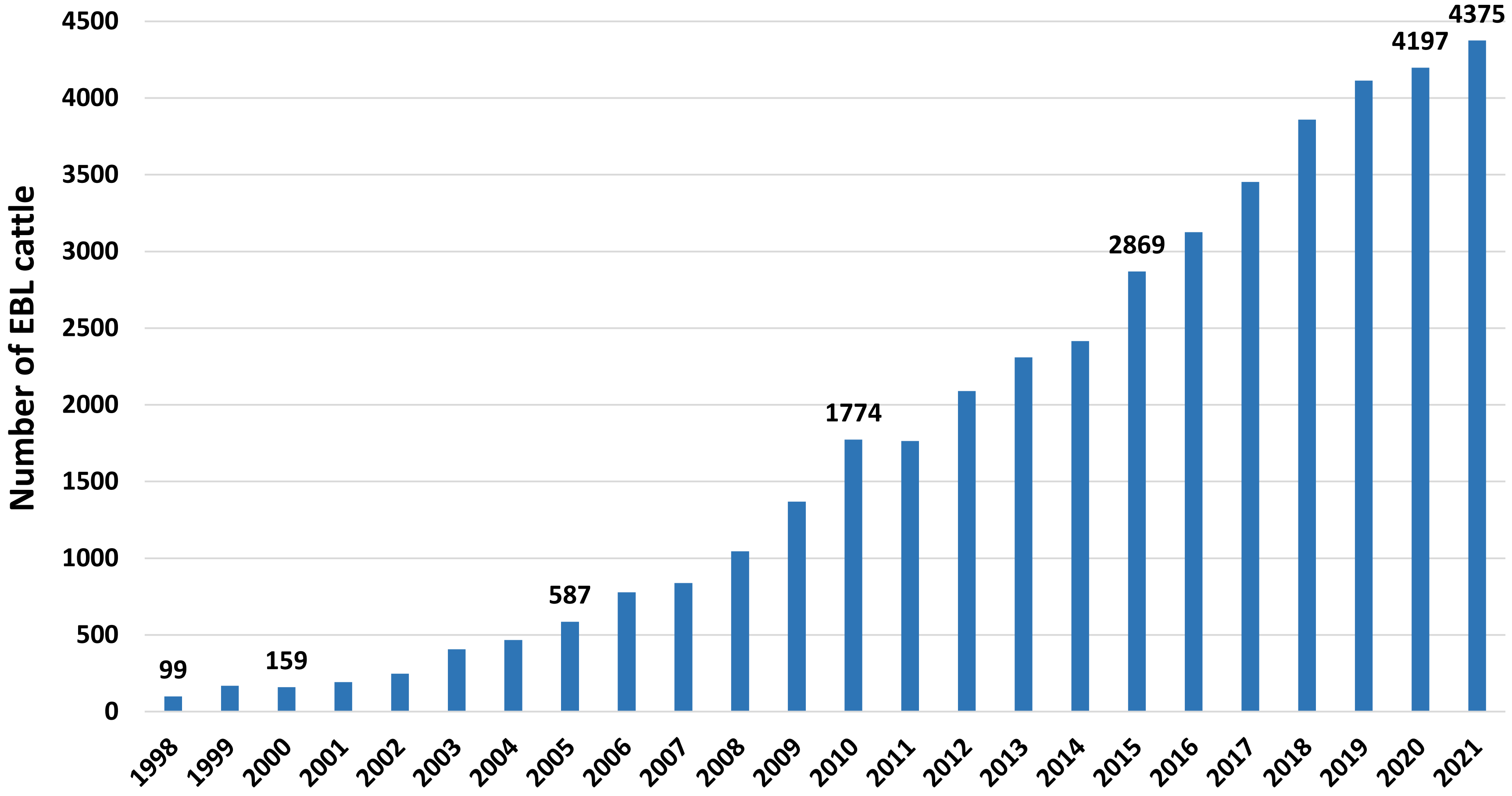

Figure S1. Number of enzootic bovine leukosis (EBL) cattle in Japan.
Data from the Surveillance of Infectious Diseases, the Ministry of Agriculture, Forestry and Fisheries, Japan. (in Japanese) Accessed 20 July, 2022. https://www.maff.go.jp/j/syouan/douei/kansi_densen/kansi_densen.html.

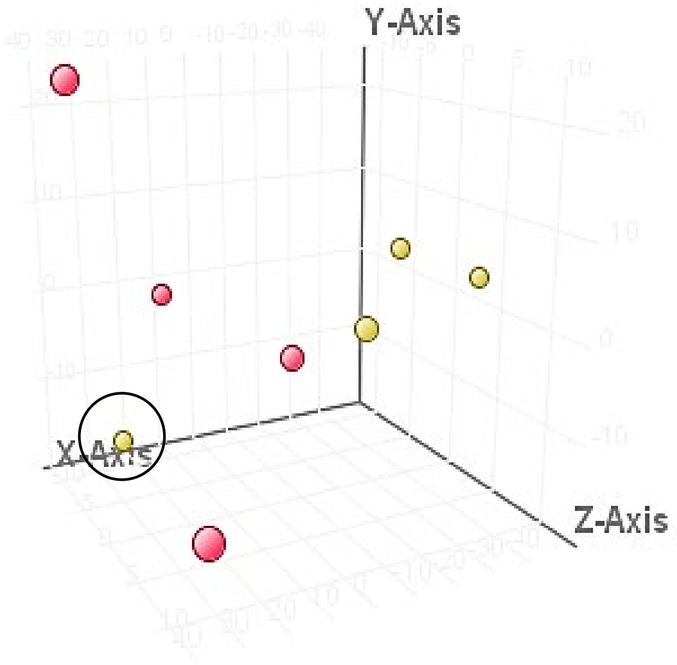

Figure S2. Principal component analysis (PCA) of miRNA amount of uninfected cattle (yellow dots) and enzootic bovine leukosis (EBL) cattle (red dots) based on microarray analysis.
The black circle shows the uninfected cow (cow no. 2 in Table 1) that was different from the other uninfected cattle.

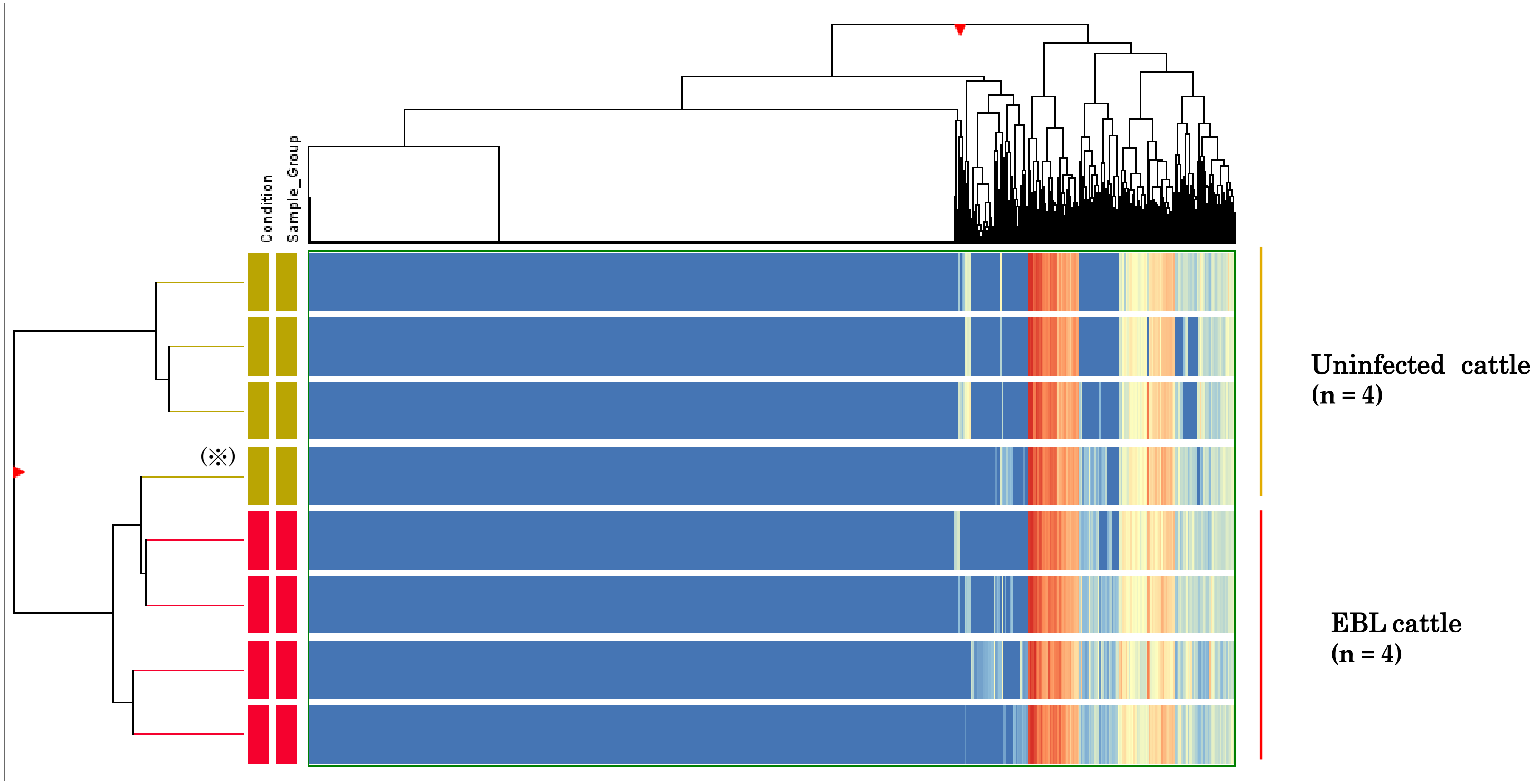

Figure S3. Cluster analysis of miRNA amount of uninfected and enzootic bovine leukosis (EBL) cattle based on microarray analysis.
One of the uninfected cattle (※) (cow no. 2 in Table 1) was classified into different cluster from the other uninfected cattle.

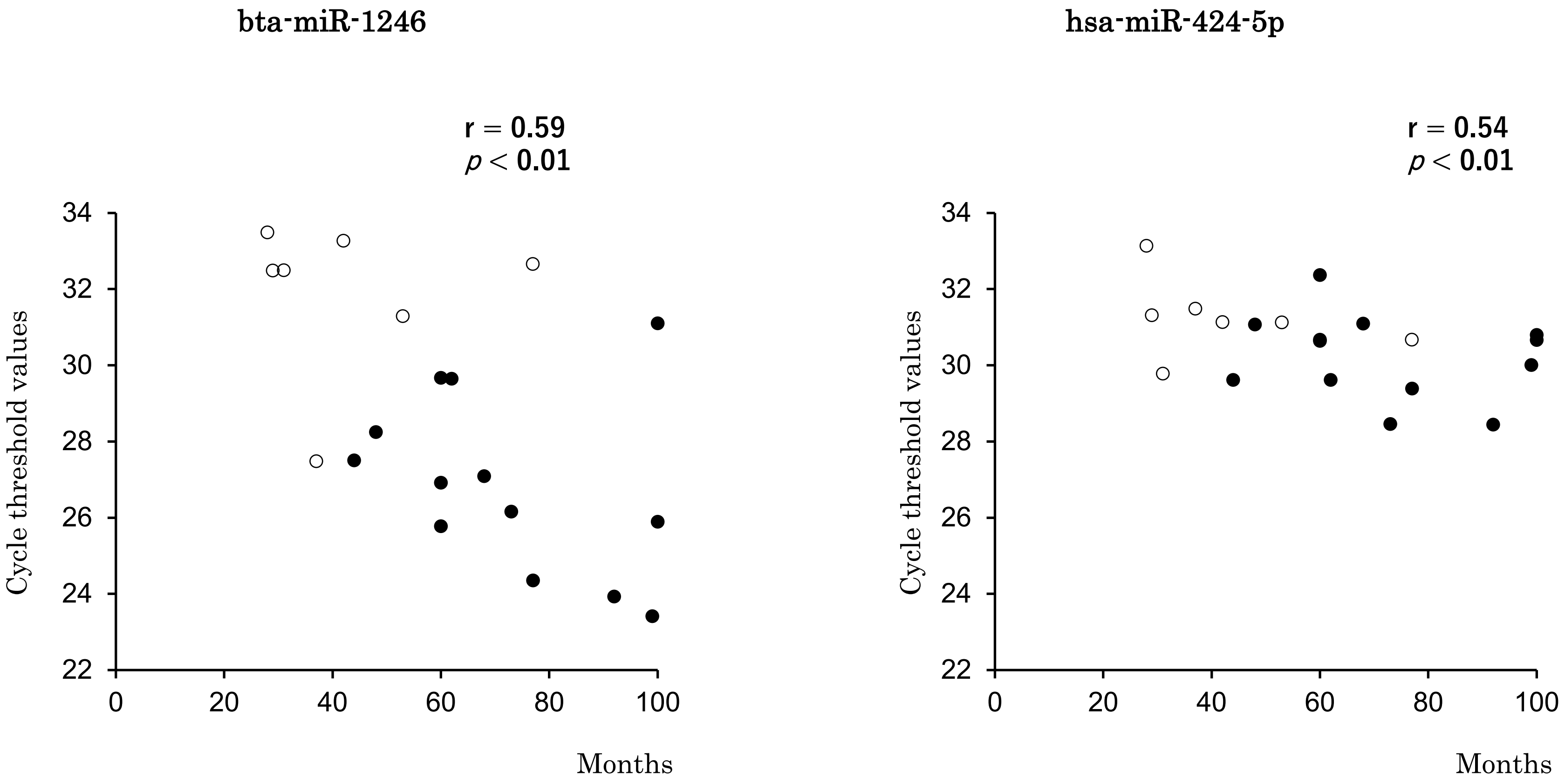

Figure S4. Correlation between age and cycle threshold values of miRNAs for biomarker candidates using milk small extracellular vesicles. The correlation between two continuous variables was determined using Spearman's bivariate correlation analysis.

○Uninfected cattle (n = 7)
●EBL cattle (n = 13)

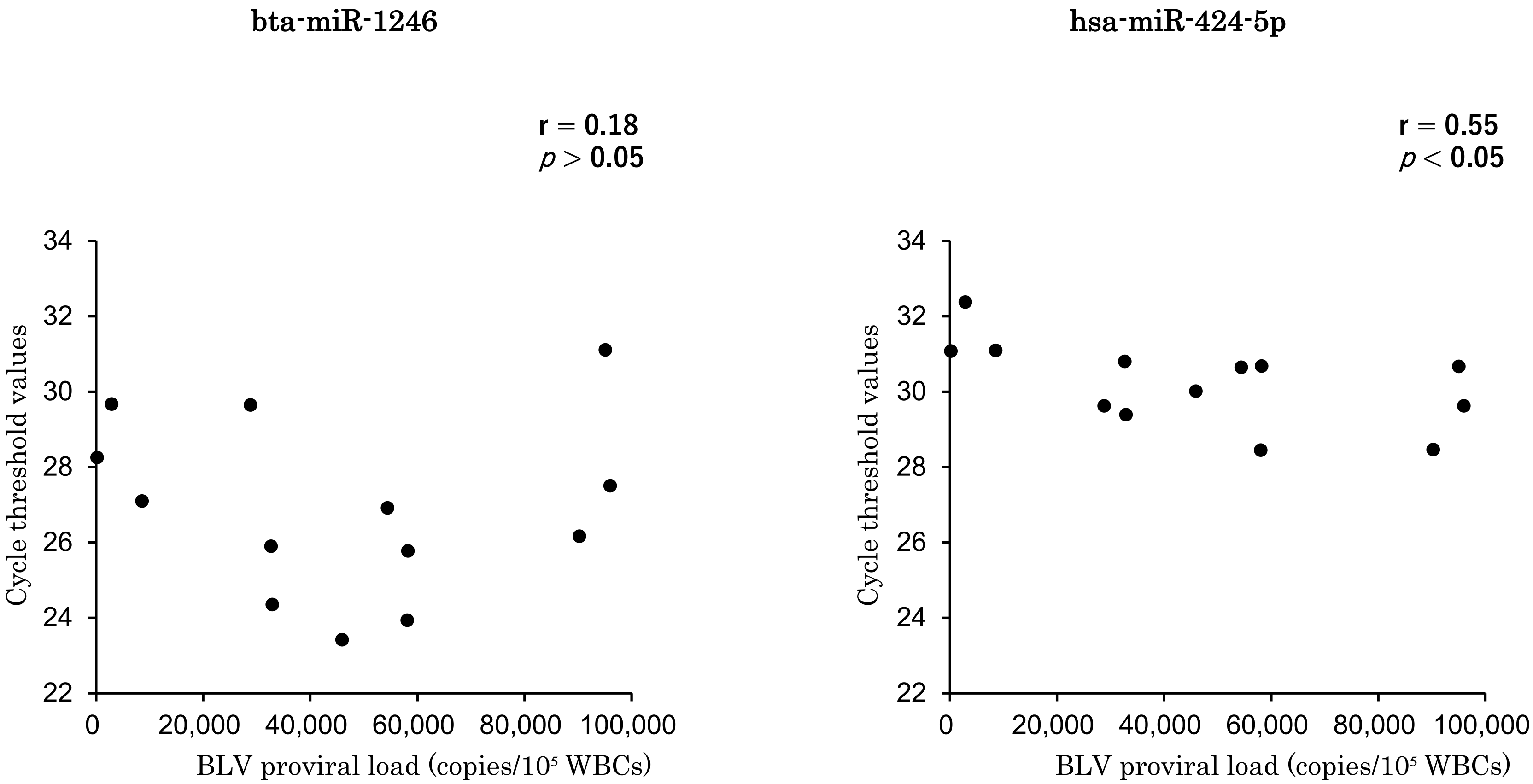

Figure S5. Correlation between bovine leukemia virus (BLV) proviral load and cycle threshold values of miRNAs for biomarker candidates using milk small extracellular vesicles. The correlation between two continuous variables was determined using Spearman's bivariate correlation analysis.

●EBL cattle (n = 13)

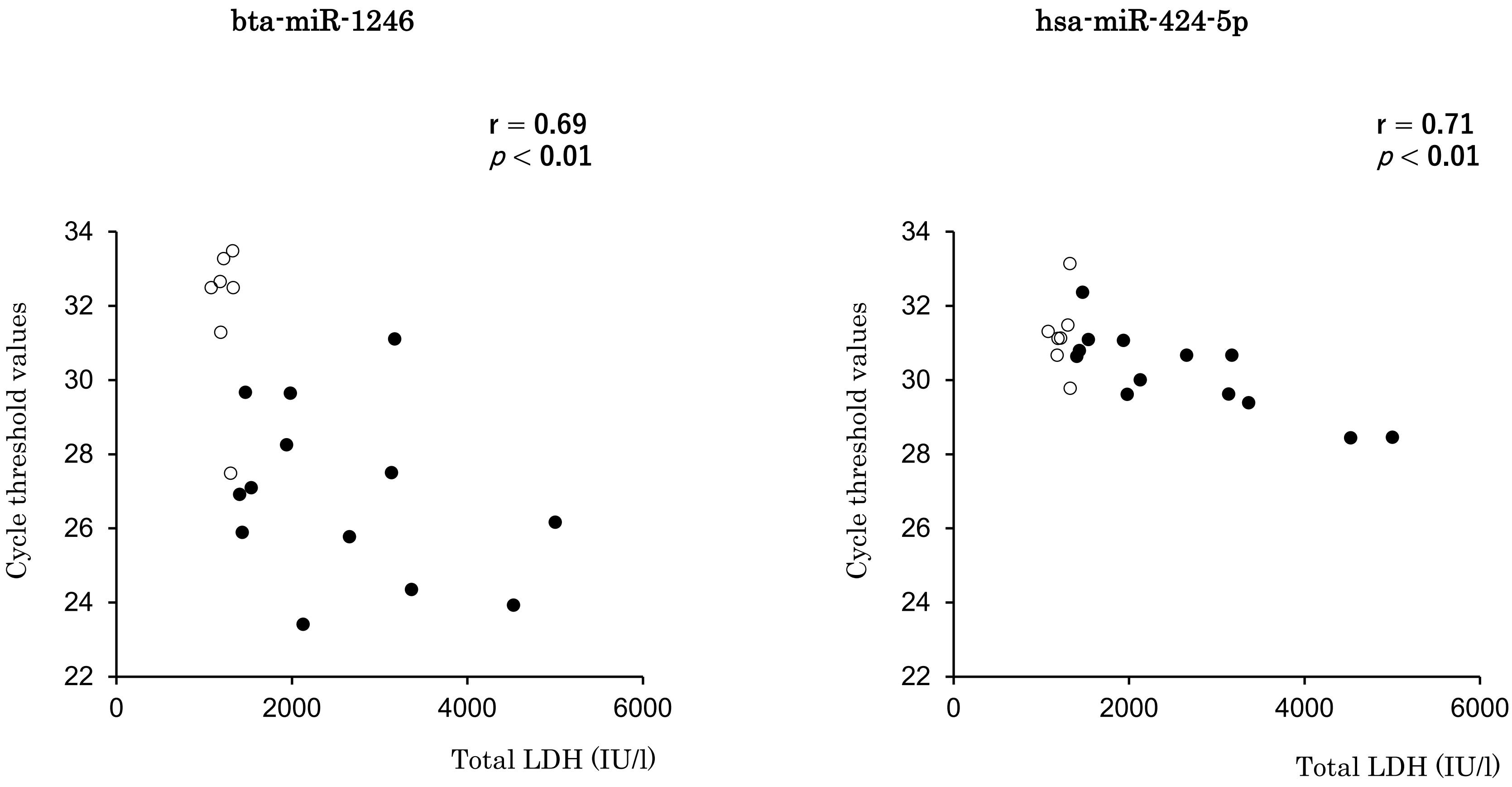

Figure S6. Correlation between total lactose dehydrogenase (LDH) activity and cycle threshold values of miRNAs for biomarker candidates using milk small extracellular vesicles. The correlation between two continuous variables was determined using Spearman's bivariate correlation analysis.

○Uninfected cattle (n = 7)
●EBL cattle (n = 13)

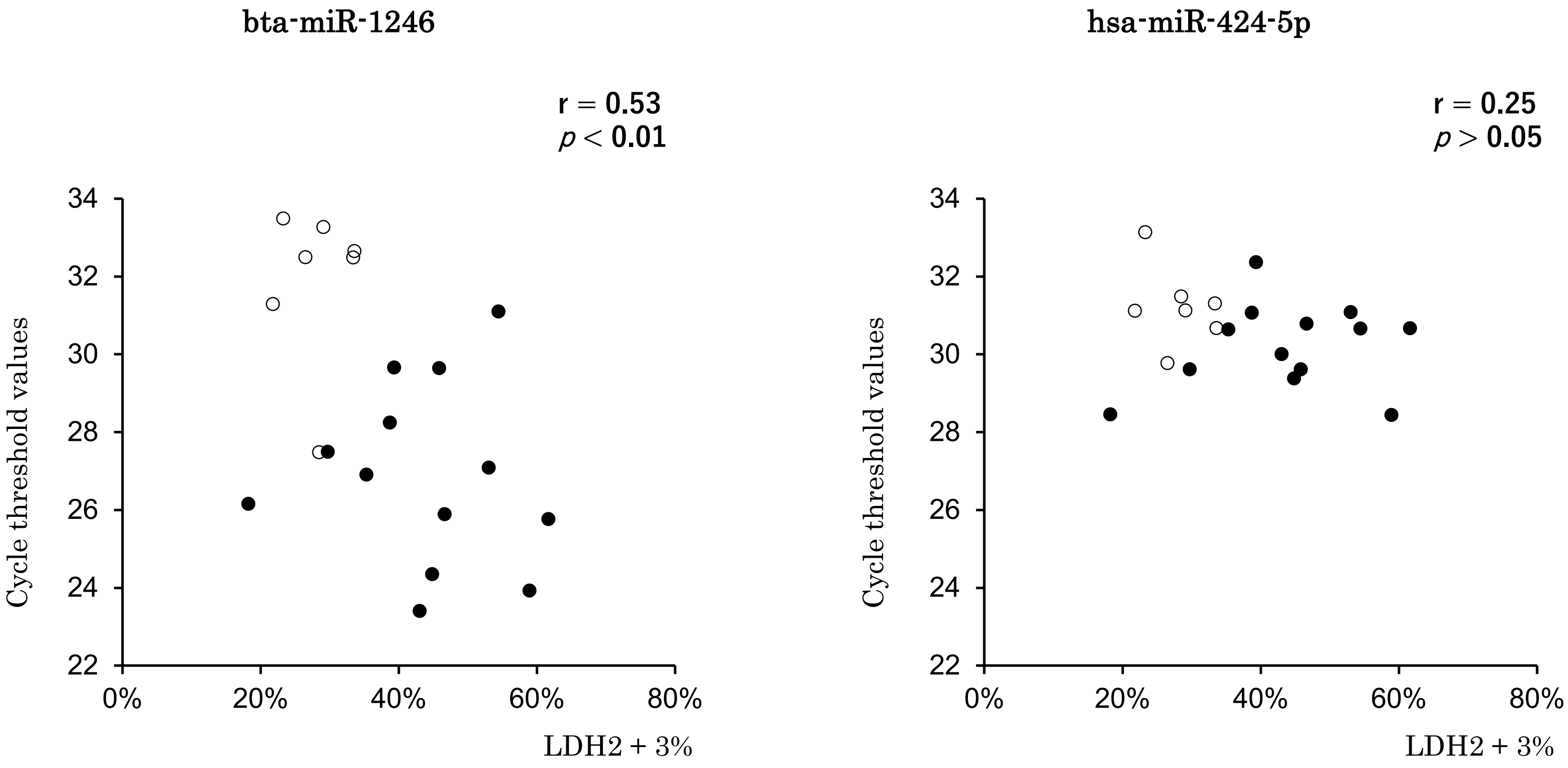

Figure S7. Correlation between lactose dehydrogenase (LDH) isozymes 2 + 3 and cycle threshold values of miRNAs for biomarker candidates using milk small extracellular vesicles. The correlation between two continuous variables was determined using Spearman's bivariate correlation analysis.

○Uninfected cattle (n = 7)
●EBL cattle (n = 13)

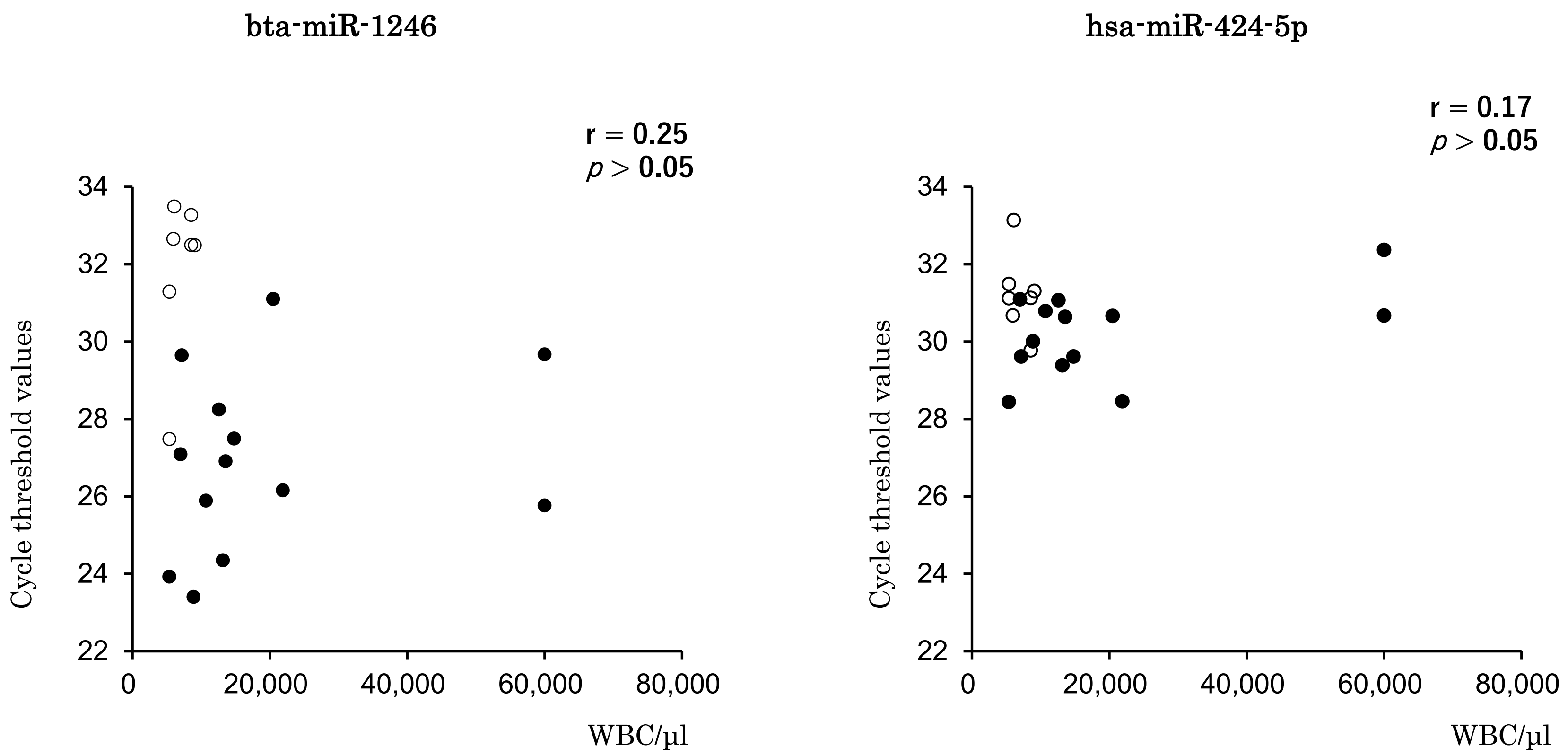

Figure S8. Correlation between white blood cell (WBC) counts and cycle threshold values of miRNAs for biomarker candidates using milk small extracellular vesicles. The correlation between two continuous variables was determined using Spearman's bivariate correlation analysis.

○Uninfected cattle (n = 7)
●EBL cattle (n = 13)

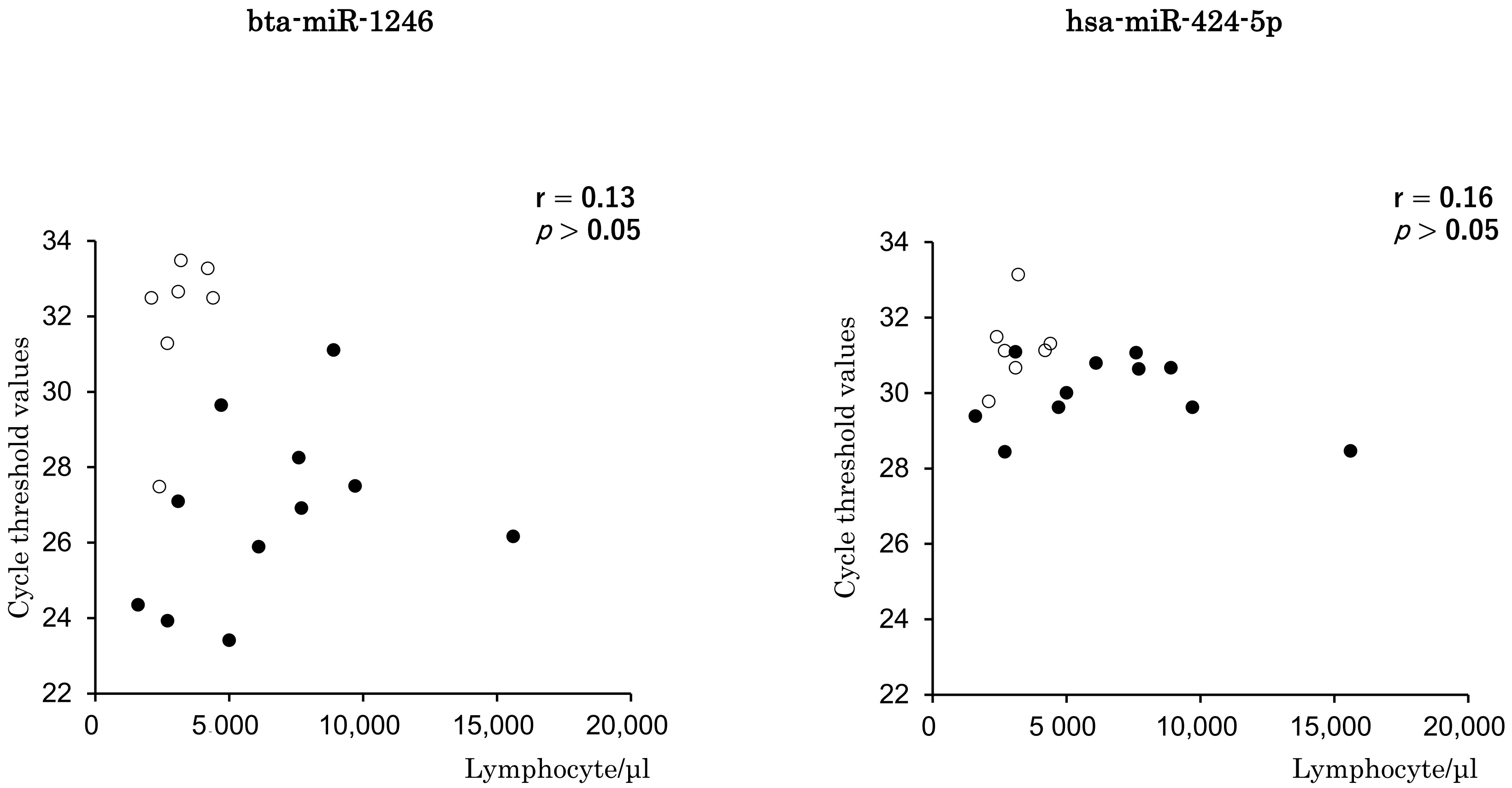

Figure S9. Correlation between lymphocyte counts and cycle threshold values of miRNAs for biomarker candidates using milk small extracellular vesicles. The correlation between two continuous variables was determined using Spearman's bivariate correlation analysis.

○Uninfected cattle (n = 7)
●EBL cattle (n = 11)
